# Production and Secretion of Isowighteone in Hairy Root Cultures of Pigeon Pea (*Cajanus cajan*) Co-Treated with Multiple Elicitors

**DOI:** 10.3390/plants11060834

**Published:** 2022-03-21

**Authors:** Gaurav Gajurel, Luis Nopo-Olazabal, Emily Hendrix, Fabricio Medina-Bolivar

**Affiliations:** 1 Arkansas Biosciences Institute, Arkansas State University, Jonesboro, AR 72467, USA; gaurav.gajurel@smail.astate.edu (G.G.); lnopo@astate.edu (L.N.-O.); emilybhendrix@gmail.com (E.H.); 2 Molecular Biosciences Graduate Program, Arkansas State University, Jonesboro, AR 72467, USA; 3 Department of Biological Sciences, Arkansas State University, Jonesboro, AR 72467, USA

**Keywords:** hairy roots, isowighteone, pigeon pea, chemical elicitors, prenylated flavonoids, methyl jasmonate, cyclodextrin, hydrogen peroxide

## Abstract

Isowighteone (3’-isoprenyl genistein) is a prenylated flavonoid derivative that exhibits antibacterial, pro-apoptotic and anti-inflammatory properties. To establish a bioproduction system for this bioactive compound, hairy root cultures of pigeon pea (*Cajanus cajan* (L.) Millsp.) were developed via *Agrobacterium rhizogenes*-mediated transformation. The cultures were co-treated with methyl jasmonate, cyclodextrin, hydrogen peroxide, and magnesium chloride to enhance the production of isowighteone. The amount of isowighteone that accumulated in the culture medium upon elicitation varied with the period of elicitation. Isowighteone was purified from extracts of the culture medium by semi-preparative HPLC, and its identity was confirmed by tandem mass spectrometry. After 144 h of elicitation in 12-day-old hairy root culture, the total yield of isowighteone was 8058.618 ± 445.78 μg/g DW, of which approximately 96% was found in the culture medium. The yield of isowighteone in the elicited hairy root culture was approximately 277-fold higher than in the non-elicited root culture. The difference between the phenotypes of the elicited and non-elicited pigeon pea hairy roots was studied using scanning electron microscopy. The non-elicited hairy roots had uniform surfaces whereas the elicited roots had non-uniform shapes. Pigeon pea hairy roots provide a sustainable platform for producing and studying the biosynthesis of isowighteone.

## 1. Introduction

Pigeon pea (*Cajanus cajan* (L.) Millsp.) is an important perennial legume crop with high medicinal and nutritional values. Extracts from pigeon pea leaves are rich in different flavonoids and stilbenoids which exhibit multiple therapeutic effects on inflammation, diabetes, dysentery, hepatitis, diarrhea, measles, and various other illnesses [1,2,3]. Several flavonoids have been reported from extracts of pigeon pea including apigenin, luteolin, isorhamnetin, vitexin, isovitexin, orientin, pinostrobin, isowighteone, and quercetin [4,5,6]. Among these flavonoids, isowighteone (3’-isoprenyl genistein) has been only reported from seedlings of pigeon pea treated with fungus and silver nitrate solution previously [5,6]. Isowighteone has shown antibacterial activity against gram-positive bacteria including *Listeria monocytogenes*, methicillin-resistant *Staphylococcus aureus*, methicillin-sensitive *Staphylococcus aureus*, and gram-negative bacteria including *Escherichia coli* [7,8]. Additionally, isowighteone has shown higher cytotoxicity against human colon carcinomas, potential pro-apoptotic property, and anti-inflammatory activities [9,10].

The extraction and purification of isowighteone from natural sources can be challenging and time-consuming, there is therefore a need to explore alternative ways to enhance its production. The Food and Agricultural Organization (FAO) has endorsed plant cell and tissue culture as an effective approach to produce phytochemicals for health and food purposes [11]. To this end, hairy root culture systems developed via *Agrobacterium rhizogenes*-mediated transformation have gained more attention recently for the stable production of various phenolics due to their genetic and biochemical stability [12]. This tissue-based system often delivers higher levels of bioactive specialized metabolites when compared to their natural sources or other plant cell/tissue culture systems [13]. Indeed, pigeon pea hairy root culture systems have been developed recently as an effective platform for the production of phenolic compounds [14].

Elicitation is one of the most effective techniques to enhance the production and accumulation of specialized metabolites in hairy root cultures [15]. Different elicitors or their combination have been used to induce the production of phenolics including stilbenoids in the hairy roots of distinct species. Co-treatment with the elicitors methyl jasmonate (MeJA), hydrogen peroxide (H_2_O_2_), and methyl-β-cyclodextrin (CD) with supplementation of magnesium chloride (MgCl_2_) has been used to enhance the production of prenylated stilbenoids in peanut (*Arachis hypogaea*) hairy root cultures [16]. Similarly, treatment with MeJA or H_2_O_2_ induces the production of stilbenoids in hairy root cultures of *Vitis rotundifolia* [17]. In previous studies, pigeon pea hairy root cultures exposed to UV irradiation led to a higher level of phenolics when compared to the non-treated control groups [18]. To our knowledge, the elicitation of pigeon pea hairy roots using chemical elicitors to increase the yield of isowighteone has not been tested previously.

This study aimed to establish a hairy root culture system for pigeon pea via *Agrobacterium rhizogenes*-mediated transformation as a sustainable production platform for isowighteone. The hairy root cultures were co-treated with a combination of MeJA, CD, H_2_O_2_, and MgCl_2_ for different periods in order to study their effect on the accumulation of isowighteone in the culture medium and hairy root tissues. Tandem mass spectroscopy was used to identify isowighteone and semi-preparative HPLC was used for its purification. Furthermore, the morphological differences between the normal and elicited hairy roots were studied using scanning electron microscopy.

## 2. Results and Discussion

### 2.1. Development and Characterization of Pigeon Pea Hairy Root Culture

**Figure 1:**
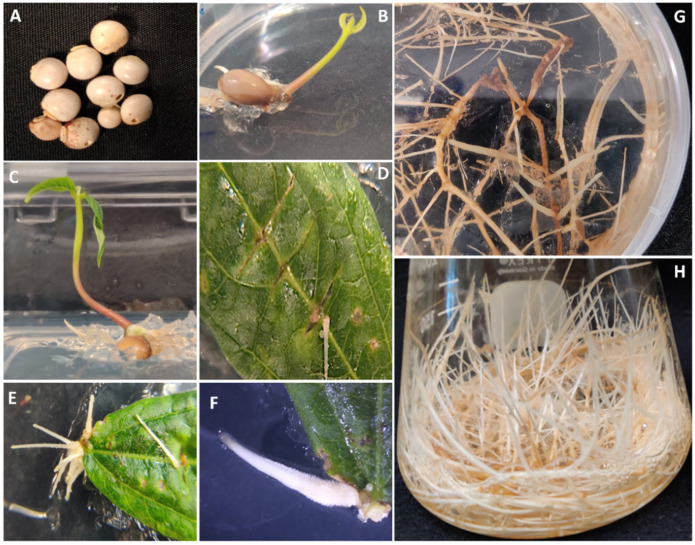
Germination and establishment of hairy root cultures of pigeon pea. (**A**): Seeds of pigeon pea; (**B**): ten-day-old seedling; (**C**): three-week-old seedling grown in vitro; (**D**–**F**): hairy root development from leaf infected with *Agrobacterium rhizogenes*; (**F**): branching of hairy roots after excision from the leaf; (**G**): phenotype of the hairy root line G4 on semi-solid medium; and (**H**): phenotype hairy root line G4 in the liquid medium after 15 days in culture.

The hairy roots of pigeon pea were induced by infecting only leaves from 3- to 4-week-old young in vitro-grown plants with *A. rhizogenes* strain K599. Hairy roots developed after approximately 3-weeks from the infection site (Figure 1A–F). Approximately ten hairy roots were harvested, and hairy root line G4 was selected for further analysis based on its sustained growth (Figure 1G). The hairy roots showed plagiotropic growth and profuse branching on semi-solid medium (Figure 1G) and liquid medium (Figure 1H). The phenotype of the hairy roots was similar to the hairy roots of another legume species, peanut, though the latter’s hairy roots exhibit a larger diameter [16]. Furthermore, PCR analysis on pigeon pea hairy root line G4 showed the presence of the *rolC* gene, confirming the integration of the T-DNA from the Ri plasmid into the plant genome. PCR validation for the *virD2* gene was negative, indicating that the hairy root tissue was *Agrobacterium*-free (Supplementary Materials Figure S1).

**Figure 2:**
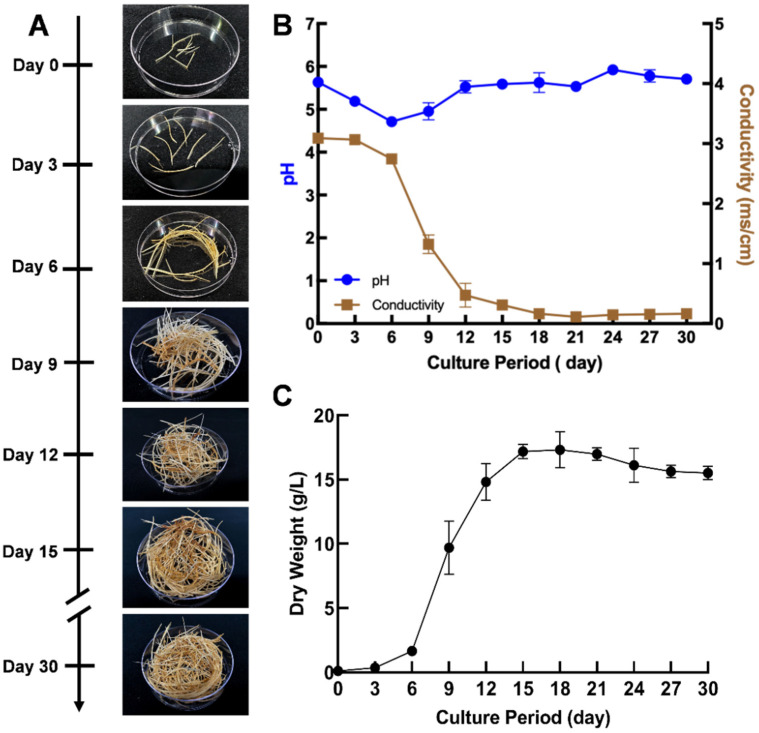
Growth of pigeon pea hairy root line G4. (**A**) Phenotype of pigeon pea hairy roots during the 30-day culture period. Pictures are representative of triplicate cultures. (**B**) Conductivity (brown) and pH of the medium (blue) at different stages of growth. (**C**) Growth curve of the hairy roots. Values represent the average of three biological replicates and the error bar represents standard deviation.

A 30-day growth curve was established to identify the growth stages of the hairy roots in liquid culture (Figure 2A). The pH and conductivity of the medium were monitored during these periods to understand nutrient utilization (Figure 2C). The conductivity of the medium decreased as the biomass increased, indicating that nutrients were taken up from the culture medium by the hairy roots during their growth. The conductivity remained constant after the hairy roots attained their highest biomass (Figure 2B). Similarly, the pH dropped for the first three days and then increased for the next three days and maintained a constant value for the remainder of the culture period (Figure 2B). Based on the dry weight of the hairy roots, a lag phase of about 3 days was observed, followed by an exponential growth phase from day 3–18 and a stationary phase from day 18–30 (Figure 2C). The pigeon pea hairy roots had a shorter exponential phase compared to the pigeon pea hairy roots described by Jiao et al., which might be due to the difference in media composition and media volume or related to the position and copy number of T-DNAs integrated into the genome [14]. The specific growth rate, growth index, and doubling times of the pigeon pea hairy root culture were calculated as 0.28 day^−1^, 154.40, and 2.47 days, respectively. The highest biomass attained was 17.30 ± 1.41 g DW/L at 18 days. This biomass yield was equivalent to other high-biomass-producing hairy root lines such as peanut, cv. Hull [13].

### 2.2. Elicitation of Hairy Root Cultures of Pigeon Pea and Identification of Isowighteone

To assess the effect of elicitation on the production of the prenylated flavonoid isowighteone, hairy root cultures of pigeon pea were treated with elicitors selected based on their efficacy on the stimulation of prenylated phenolics in hairy root cultures of peanut. Among the elicitors, MeJA has been widely used as a signaling molecule to induce the biosynthesis of low molecular weight phytoalexins, such as stilbenoids. CD has been used as an elicitor and trapping agent to prevent the feedback inhibition of inducible metabolites, whereas H_2_O_2_ is a signaling molecule that induces oxidative stress and stimulates the production of specialized metabolites [19]. For instance, black carrot (*Daucus carota* L. ssp. *sativus* var. *atrorubens* Alef) hairy roots treated with H_2_O_2_ led to a controlled oxidative burst, resulting in the increased production of anthocyanins by almost 20% [20]. Magnesium (Mg^2+^) supplementation has been used for its role as a co-factor for the prenyltransferase enzymes involved in the prenylation of phenolics compounds [21,22].

Notably, after 96 h of elicitation treatment, the color of the medium started to change from clear to yellowish, suggesting the induction and accumulation of prenylated phenolics in the culture medium. A similar yellow color was observed when peanut hairy root cultures were co-elicited to produce the prenylated stilbenoids arachidin-1 and arachidin-3 [23]. Based on these observations, the culture medium was initially analyzed for the presence of isowighteone. HPLC analyses of the ethyl acetate extract of the culture medium show 5 major peaks with Rts of 9.44, 26.83, 28.51, 31.84, and 40.28 min.

**Figure 3:**
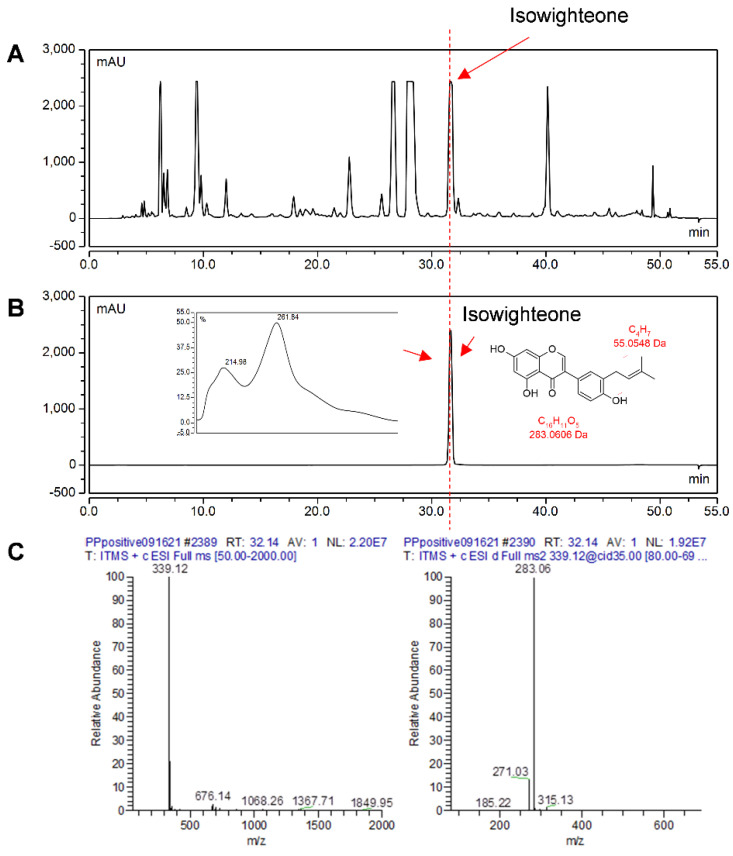
Purification of isowighteone. (**A**) HPLC chromatogram of ethyl acetate extract from the medium of pigeon pea hairy root culture. (**B**) HPLC chromatogram of purified isowighteone with its UV spectrum and chemical structure. All chromatograms were monitored at 260 nm. (**C**) HPLC-PDA-electrospray ionization-MS^2^ analysis of isowighteone (left: MS; right: MS^2^).

The 144 h extract was further analyzed by tandem mass spectrometry and the identity of the peak with an Rt of 31.84 was confirmed as isowighteone by comparison to UV spectrum (λ_max_ = 261 nm) and mass spectroscopy data previously reported for isowighteone [23]. As shown in Table 1, the precursor ion of the targeted compound ([M + H]^+^, *m*/*z* 339) provided a daughter ion with a [M + H]^+^ of *m*/*z* 283 in MS^2^, suggesting the removal of the prenyl group and corroborating the identification of isowighteone in the elicited culture medium [24] (Supplementary Materials Figure S2). In order to obtain sufficient material for quantitative analyses, the media from several 144-h-elicited cultures were pooled and extracted with ethyl acetate. The extract was further separated by semi-preparative HPLC to yield isowighteone with >95% purity (Figure 3). The latter was used to establish a calibration curve for isowighteone.

### 2.3. Scanning Electron Microscopy of Non-Elicited and Elicited Hairy Roots

**Figure 4:**
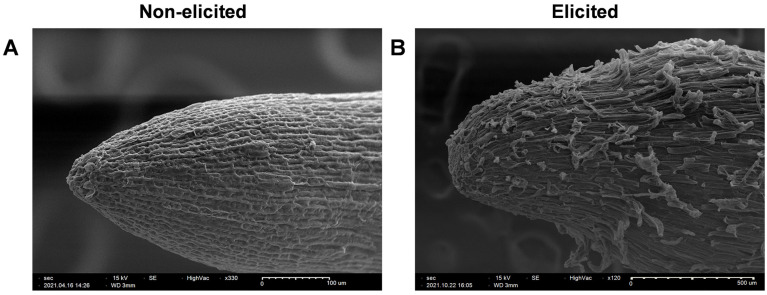
Scanning electron microscopy analysis of hairy roots of pigeon pea line G4: (**A**) Non-elicited under ×330 magnification and (**B**) elicited for 144 h under ×120 magnification.

To stimulate the production of specialized metabolites, pigeon pea hairy root cultures were treated with elicitors. The non-elicited hairy roots continued to grow, whereas growth slowed down in the elicited hairy roots. After 144 h of the elicitation period, the detailed structure of the tips of the non-elicited and elicited hairy root tips were observed using scanning electron microscopy (SEM). Previously, SEM has been used to analyze the morphology of hairy roots of pigeon pea, *Scutellaria lateriflora*, and *Catharanthus roseus* [18,25,26]. In this study, morphological changes were observed between the elicited and non-elicited hairy roots of pigeon pea (Figure 4). The cells in the non-elicited hairy root tips were tube-like and smooth. Interestingly, the cells in elicited hairy root tips seemed to have a non-uniform shape along with some ruptures on the surface. The elicited roots appeared to have a blunt end and several layers of the tissue were detached from the surface. A similar phenomenon was reported in hairy roots of pigeon pea exposed to UV B radiation and *Catharanthus* hairy roots treated with MeJA, where roots were mostly damaged after treatment [18,26].

### 2.4. Time Course Accumulation of Isowighteone in the Culture Medium

**Figure 5:**
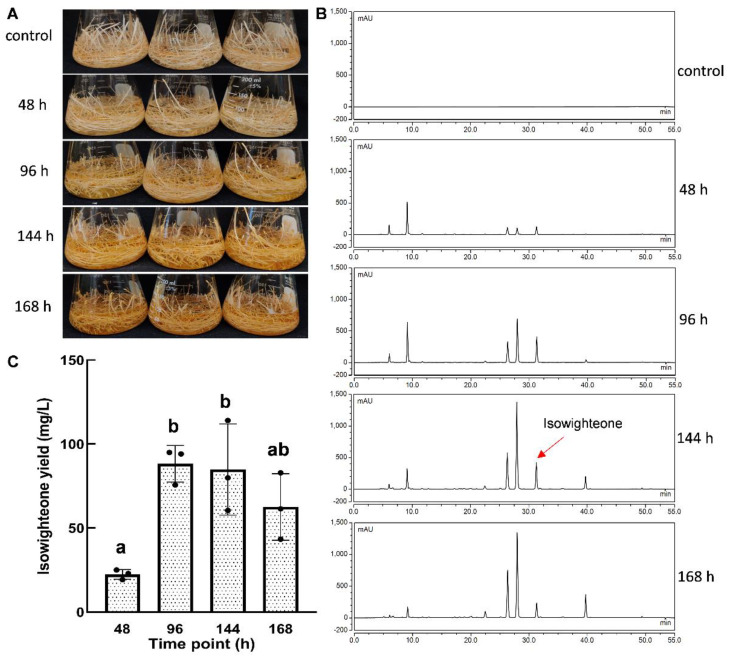
Time course of isowighteone yield in the elicited culture medium of 12-day-old pigeon pea hairy root cultures. (**A**) Change in phenotype after elicitor treatment. (**B**) HPLC chromatogram of culture medium extract after elicitor treatment for different time points. All chromatograms were monitored at 260 nm. (**C**) Comparison of isowighteone yield in the culture medium of hairy root cultures elicited for different time points. Yields are expressed in mg/L. Each bar represents the average of three biological replicates. Error bar represents standard deviation. Statistical analysis was performed using one-way ANOVA with Tukey’s multiple comparisons test. The lower-case letters above the column represent significant (between different letters) or non-significant (between the same letter) statistical differences. (Significance level between a and b, *p* < 0.05).

Based on previous elicitation studies with prenylated stilbenoids [16,23] and the above experiment, 12-day-old hairy root cultures (mid-log stage) were selected and elicited for different time periods, i.e., 48 h, 96 h, 144 h, and 168 h. The yellowish color of the medium increased gradually with the elicitation time and the highest intensity was observed at 168 h (Figure 5A). HPLC analysis of the ethyl acetate extract from the culture medium showed a higher accumulation of isowighteone as the elicitor treatment time increased to 144 h (Figure 5B). In the current study, the yields of isowighteone in the culture medium after 48 h, 96 h, 144 h, and 168 h of elicitation were 22.43 ± 2.88, 88.24 ± 10.93, 84.79 ± 27.16, and 62.51 ± 19.90 mg/L, respectively. The yields of isowighteone in hairy root cultures elicited for 96 h and 144 h were significantly higher than those elicited for 48 h (Figure 5C).

### 2.5. Yield of isowighteone in the Hairy Root Culture System

**Figure 6:**
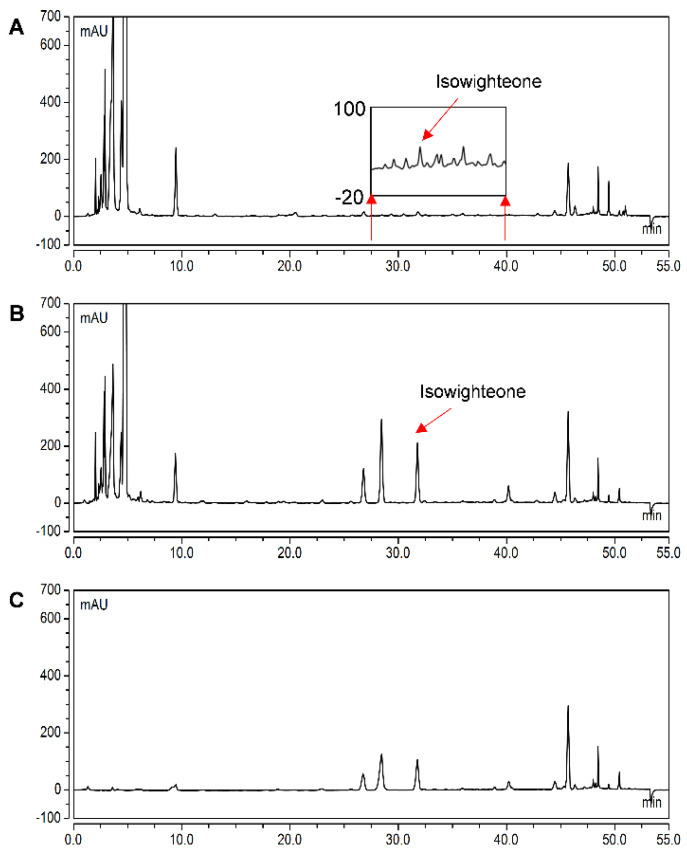
Comparison of isowighteone yield in hairy root tissues of 12-day-old pigeon pea hairy roots. HPLC chromatogram of (**A**) Non-elicited hairy root tissue extracted using 70% ethanol; (**B**) 144 h-elicited hairy roots extracted using 70% ethanol; and (**C**) 144 h-elicited hairy roots extracted using ethyl acetate. All chromatograms were monitored at 260 nm.

To assess the overall yield of isowighteone in the pigeon pea hairy root culture system, 12-day-old cultures were selected and elicited for 144 h. Intriguingly, isowighteone was detected in the elicited culture medium, elicited hairy root tissue, and non-elicited hairy root tissue. However, the majority of isowighteone was found in the elicited culture medium. To determine the ratio of isowighteone present in the culture medium versus hairy root tissue, the yields were determined per g DW of the root. As shown in Table 2, the yield of isowighteone in the elicited hairy root tissue extracted with ethanol was 346.35 ± 63.65 μg/g DW whereas the yield of isowighteone using ethyl acetate as the extraction solvent was 231.81 ± 32.73 μg/g DW (Figure 6). Thus, the recovery of isowighteone from root tissue was higher when ethanol was used as a solvent for extraction. The yield of isowighteone in the elicited culture medium was 7712.27 ± 441.23 μg/g DW. The combined yield of isowighteone in the medium and root tissue of the 12-day-old culture elicited for 144 h was 8058.62 ± 445.78 μg/g. To our knowledge, this is the highest level of isowighteone reported yet. Strikingly, in the current study, the total yield of isowighteone in elicited hairy root culture system was approximately 277-fold higher than under non-elicited conditions. After the elicitation of the pigeon pea hairy root culture, almost 96% of the total isowighteone was secreted into the culture medium. Furthermore, the elicitation procedure enhanced the secretion of most of the isowighteone into the culture medium, thereby facilitating the extraction and purification of this biologically active important compound from pigeon pea hairy root cultures.

In this study, several other compounds with a wavelength similar to isowighteone were detected. The hairy root system can be further used to purify and confirm the structure of other phenolics induced upon elicitor treatment. Previously, a hairy root system treated with elicitors was used to explore the biosynthetic pathway of stilbenoids in peanuts [27]. Similarly, pigeon pea hairy root may provide a platform to elucidate the biosynthetic pathway of isowighteone and related phenolics.

## 3. Materials and Methods

### 3.1. Seed Sterilization and Germination of Pigeon Pea

Pigeon pea seeds (*Cajanus cajan* (L.) Millsp.; SKU: GK-J9X6-0NKT) were sourced in south Florida (USA) and obtained from Palm Beach Medicinal Herbs (West Palm Beach, FL, USA). The seeds were surface-sterilized by soaking in 0.1% Palmolive^®^ detergent for 2 min followed by vigorous shaking in 50% Clorox^®^ for 15 min. Then, the seeds were rinsed using sterile distilled water 3–4 times and cultured in 100 mm × 20 mm Petri Dishes (Fisherbrand^®^, Fisher Scientific™, Hampton, NH, USA), with one seed per Petri Dish, containing Murashige and Skoog (MS) medium with 3% sucrose (pH 5.7) and 0.4% phytagel in dark conditions until germination. After germination, the seeds were transferred to Phytatray™ boxes (Millipore Sigma, St. Louis, MO, USA) and kept in a photoperiod incubator (16 h light/8 h dark) at 24 °C for further growth.

### 3.2. Establishment of Hairy Root Lines of Pigeon Pea

Leaves were excised from in vitro 3–4-weeks-young plants of pigeon pea and inoculated with *Agrobacterium rhizogenes* strain K599 maintained on LB solid media (Fisher Scientific™, Hampton, NH, USA) using a scalpel. The leaves were then placed on MSV medium [13] and incubated for 5–7 days till *Agrobacterium* growth was observed on the leaves [13]. The leaves were then transferred to MSV semi-solid medium with 250 mg/L cefotaxime until hairy roots were developed. Next, the hairy roots were excised from the leaves, placed on MSV semi-solid medium with 250 mg/L cefotaxime for 2–3 weeks, and then transferred to MSV semi-solid medium without any antibiotics for further growth. Pigeon pea hairy root line G4 was selected among several hairy roots for further analysis due to its vigorous and sustained growth. To confirm the establishment of hairy roots, genomic DNA was extracted using the DNeasy Plant Mini kit (Qiagen, Germantown, MD, USA), and PCR analysis for *rolC* and *virD2* genes was performed, as described before [28].

### 3.3. Growth Kinetics of Pigeon Pea Hairy Root Line

A total of ten 2–3 cm long tips were excised from the hairy roots growing in MSV semi-solid medium and cultured in a 250 mL flask containing 50 mL of MSV liquid medium with 3% sucrose to establish a growth curve for hairy root line G4 [13]. The flask cultures were covered with aluminum foil and maintained on an orbital shaker (Innova 44R, New Brunswick Scientific, Hauppauge, NY, USA) at 90 rpm and 28 °C in continuous darkness. Three flasks were harvested at intervals of 3 days through to day 30. The harvested hairy roots were rinsed with tap water and lyophilized (Freeze Dry System Freezone 4.5, Labconco™, Kansas City, MO, USA) to obtain the dry weight. The specific growth rate (µ) was calculated as

$$µ=ln\;\left(DW_{i}/DW_{0}\right)/\Delta t$$

where *DW_i_* = 17.30 g/L is the average dry weight of the roots in grams at the end of the exponential growth (day 18), *DW_0_* = 0.11 g/L is the average dry weight of inoculum in grams at the start of the exponential growth (day 0), and *t* is the interval of time (in days) between *0* and *i* (18 days). Doubling time (T_d_) was calculated as

$$T_{d}=ln\left(2\right)/µ$$

where µ is the calculated specific growth rate of the hairy root.

The growth index (GI) was calculated as

$$\mathrm{GI}=\left(DW_{i}-DW_{0}\right)/DW_{0}\;$$

where *DW_i_* is the average dry weight of the roots in grams at the end of the exponential growth (day 18) and *DW_0_* is the average dry weight of inoculum in grams at the start of the exponential growth (day 0). The conductivity and pH were measured from the culture media at each of the time points.

### 3.4. Elicitation of Pigeon Pea Hairy Root Cultures

Hairy roots of pigeon pea were cultured in a 250 mL flask with 50 mL of MSV medium for 12 days before elicitation. The spent medium was discarded and replaced with elicitation medium, i.e., MSV medium [13] containing 3% sucrose with 125 μM methyl jasmonate (MeJA; Sigma-Aldrich, St. Louis, MO, USA), 18 g/L methyl-β-cyclodextrin (CD; CAVASOL^®^ W7 M, Wacker, Munich, Germany), 3 mM hydrogen peroxide (H_2_O_2_; Thermo Scientific, Waltham, MA, USA), and 1 mM magnesium chloride (MgCl_2_; Sigma-Aldrich, St. Louis, MO, USA) [16]. The elicitation was carried out in continuous darkness for 144 h (6 days) at 28 °C. To optimize the elicitation time point, day 12 pigeon pea hairy root cultures were elicited under conditions as described above and an aliquot of 900 µL was taken at 48, 96, 144, and 168 h for further analysis.

### 3.5. Extraction and Analysis of Phenolic Compounds

To extract phenolics from the culture medium, a 900 µL aliquot from the elicited culture medium was mixed with 900 µL of ethyl acetate by vortexing for 30–45 s. A volume of 500 µL of the upper organic phase was removed and transferred to an HPLC vial and then dried under a nitrogen stream using a Reacti-Vap III apparatus (Thermo Scientific, Waltham, MA, USA). The extract was resuspended in 500 µL methanol and analyzed using an HPLC system, as detailed below.

For tissue extraction, 12-day-old hairy roots elicited for 144 h were lyophilized for 48 h and 100 mg of lyophilized tissue was extracted with two different solvents, i.e., 2 mL of 70% aqueous ethanol solution and 2 mL of ethyl acetate in an ultrasonic bath for 8 min followed by centrifugation. The supernatant was collected and analyzed by HPLC, as described below. HPLC analysis was performed using a Sunfire™ C18, 5 µm, 4.6 × 250 mm column (Waters, Milford, MA, USA) at 40 °C, and a flow rate of 1.0 mL/min. The mobile phase consisted of methanol (A) and 0.5% formic acid (B). A linear gradient was performed from 55% A to 65% A for 0–20 min, then from 65% A to 85% A for 20–43 min, from 85% A to 100% A for 43–50 min, and 100% A to 55% A for 50–55 min. A dilution of purified isowighteone (>95% purity determined by HPLC at 260 nm) was made in methanol to obtain a calibration curve for quantification. A calibration curve for isowighteone was established at A_260_ (y = 1.07x + 0.3941, LOQ = 16.529 mg/L, LOD = 4.959 mg/L, R^2^ = 0.9996) using isowighteone purified from the hairy root culture medium, as described below.

Liquid chromatography-mass spectrometry qualitative analysis of isowighteone was performed using an UltiMate 3000 rapid separation LC system (Thermo Scientific, Waltham, MA, USA). The HPLC conditions for separation are described above. The LTQ XL linear ion trap mass spectrophotometer (Thermo Scientific, Waltham, MA, USA) with an electrospray ionization source was used for obtaining structural information relating to isowighteone following the method described previously [25]. Briefly, full scans were recorded in the mass range of *m*/*z* 50 to 2000. The mass spectra analysis was performed in positive and negative modes with the capillary temperature at 300 °C, sheath gas at 45 arbitrary units, and ion spray voltage at 4 kV. A collision energy of 35% was applied in collision-induced dissociation and ultra-high-purity helium gas was used as the collision gas. The data was recorded and analyzed by Xcalibur software (Thermo Scientific, Waltham, MA, USA).

### 3.6. Identification and Purification of Isowighteone

For the purification of isowighteone, 1 L of the 144 h-elicited medium was pooled from 10 flasks of pigeon pea hairy root cultures. The medium was partitioned with an equal volume of ethyl acetate twice in a 2-L separatory funnel. The obtained organic phase was recovered and dried in a rotavapor (Büchi, rotavapor R-2000, St. Gallen, Switzerland), and the extract (approximately 1.47 g) was further used for semi-preparative HPLC.

For semi-preparative HPLC, a Sunfire^®^ C18 OBD™ Prep, 10 × 250 mm column (Waters, Milford, MA, USA) was used. Chromatography was performed at 40 °C and a flow rate at 4.0 mL/min. The mobile phase consisted of methanol (A) and 0.5% formic acid (B). A linear gradient was performed from 55% A to 65% A for 0–20 min, then from 65% A to 85% A for 20–43 min, from 85% A to 100% A for 43–50 min, and 100% A to 55% A for 50–55 min. Based on retention time and UV, the isowighteone peak was collected and dried under nitrogen gas. The peak was validated as isowighteone using tandem mass spectroscopy, as described above.

### 3.7. Scanning Electron Microscopy

Morphological analysis of 12-day-old hairy roots elicited for 144 h and non-elicited 12-day-old hairy roots were performed using scanning electron microscopy (SEM) (SNE-4500 plus, NanoImages, Pleasanton, CA, USA). The hairy root tissue was fixed as described before [25]. Briefly, primary fixation was performed using 2% glutaraldehyde in phosphate buffer saline for 1 h at room temperature, followed by three PBS washes of 10 min each. Secondary fixation was completed by treating the tissues in 1% osmium tetraoxide in PBS for 1 h at room temperature. The tissue was washed three times with water for 15 min. The hairy roots were then dehydrated by processing them through a series of ethanol washes to ensure that water was removed. The tissue was processed in a critical point dryer and then mounted on aluminum SEM stubs with double-stick conductive carbon tape. Stubs with tissue were coated with gold in a sputter coater machine (MCM-100P, NanoImages, Pleasanton, CA, USA) and viewed under SEM. The SEM was operated at a high vacuum at 15 kV.

### 3.8. Statistical Analysis

One-way ANOVA with Tukey’s multiple comparison tests was performed for Figure 5 with GraphPad Prism 9 software, version 9.30 (San Diego, CA, USA).

## 4. Conclusions

In conclusion, hairy roots cultures of pigeon pea were developed and co-treated with a combination of the elicitors methyl jasmonate, methyl-β-cyclodextrin, hydrogen peroxide, and supplemented with magnesium chloride to enhance the production of isowighteone. The treatment of hairy roots with optimized elicitation conditions led to a high yield of isowighteone into the culture medium and root tissues. Also, the yield of isowighteone varied with the time of elicitor treatment. In addition, scanning electron microscopy imaging suggested that the hairy roots were stressed upon elicitor treatment. In the future, this sustainable platform could aid in elucidating the biosynthetic pathway of isowighteone and other related phenolics in pigeon pea. Moreover, the hairy roots may provide an alternative platform for the extraction and purification of isowighteone for further exploration of its bioactivity in vivo.

## Figures and Tables

**Table 1:** Mass spectrometry analysis of isowighteone detected in ethyl acetate extract from the medium of elicited pigeon pea hairy root culture. Analysis was done by HPLC-PDA-electrospray ionization-MS^2^.

*t*_R_ (min)	UV Max (nm)	[M − H]^−^	MS^2^ Ions	[M + H]^+^	MS^2^ Ions
31.84	261	337	268, 281, 294	339	283, 271, 255

*t*_R_: Retention time in minutes.

**Table 2:** Yield of isowighteone in the hairy root culture of pigeon pea (line G4). Twelve-day-old cultures were co-treated with methyl jasmonate, methyl-β-cyclodextrin, hydrogen peroxide, and magnesium chloride.

Source	Yield of isowighteone (μg/g DW)	
	Control	Elicitor Treatment
Hairy root tissue	29.08 ± 3.7	346.35 ± 63.65
Culture medium	bDL ^a^	7712.27 ± 441.23
Total yield of isowighteone	29.08 ± 3.7	8058.618 ± 445.78

^a^ Below Detection Limit.

## Data Availability

Data is contained within the article and supplementary material.

## Supplementary Materials

The following supporting information can be downloaded at: https://www.mdpi.com/2223-7747/11/6/834/s1, Figure S1: PCR analysis of pigeon hairy root line G4 with primers targeting the rolC and virD2 genes. Plasmid pRiK599 was used as the positive control and ddH_2_O was used as the negative control; Figure S2: MS ion chromatogram of isowighteone under negative mode.
