# Correction: Gajurel et al. Production and Secretion of Isowighteone in Hairy Root Cultures of Pigeon Pea (*Cajanus cajan*) Co-Treated with Multiple Elicitors. *Plants* 2022, *11*, 834

**DOI:** 10.3390/plants11192665

**Published:** 2022-10-10

**Authors:** Gaurav Gajurel, Luis Nopo-Olazabal, Emily Hendrix, Fabricio Medina-Bolivar

**Affiliations:** 1 Arkansas Biosciences Institute, Arkansas State University, Jonesboro, AR 72467, USA; 2 Molecular Biosciences Graduate Program, Arkansas State University, Jonesboro, AR 72467, USA; 3 Department of Biological Sciences, Arkansas State University, Jonesboro, AR 72467, USA

In the original publication [1], there was a mistake in the Title, Table 1, Figure 3, Figure 5, Table 2, and Figure 6, as published. The name of the compound cajaninstilbene acid was not correct and has been replaced with isowighteone. This error resulted from the misidentification of isowighteone as cajaninstilbene acid in previous publications. 

The corrected article title should be “Production and Secretion of Isowighteone in Hairy Root Cultures of Pigeon Pea (*Cajanus cajan*) Co-Treated with Multiple Elicitors”.

The corrected Table 1, Figure 3, Figure 5, Table 2, and Figure 6 appear below.

**Table 1:** Mass spectrometry analysis of isowighteone detected in ethyl acetate extract from the medium of elicited pigeon pea hairy root culture. Analysis was performed with HPLC-PDA-electrospray ionization-MS^2^.

*t*_R_ (min)	UV Max (nm)	[M − H]^−^	MS^2^ Ions	[M + H]^+^	MS^2^ Ions
31.84	261	337	268, 281, 294	339	283, 271, 255

*t*_R_: Retention time in minutes.

**Figure 3:**
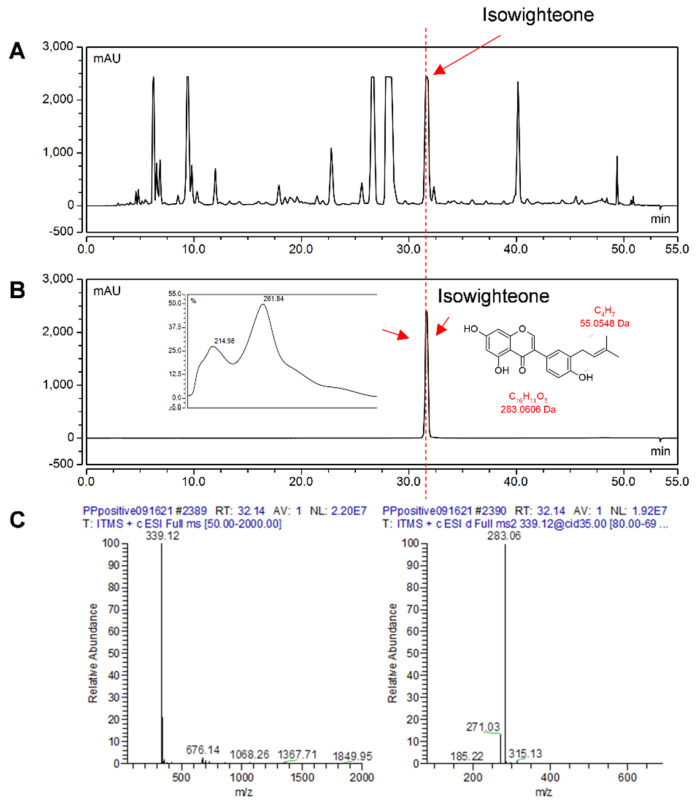
Purification of isowighteone. (**A**) HPLC chromatogram of ethyl acetate extract from the medium of pigeon pea hairy root culture. (**B**) HPLC chromatogram of purified isowighteone with its UV spectrum and chemical structure. All chromatograms were monitored at 260 nm. (**C**) HPLC-PDA-electrospray ionization-MS*^2^* analysis of isowighteone (left: MS; right: MS^2^).

**Figure 5:**
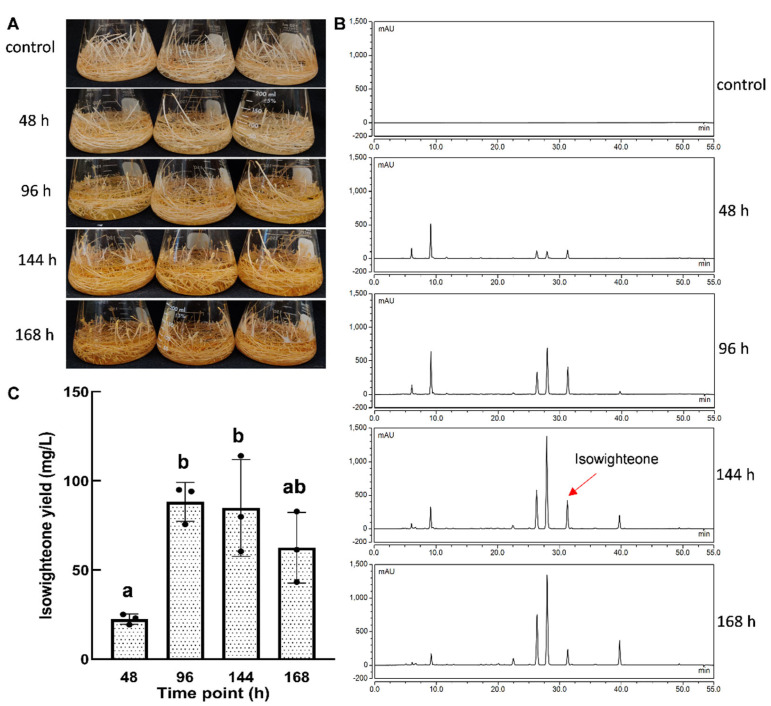
Time course of isowighteone yield in the elicited culture medium of 12-day-old pigeon pea hairy root cultures. (**A**) Change in phenotype after elicitor treatment. (**B**) HPLC chromatogram of culture medium extract after elicitor treatment for different time points. All chromatograms were monitored at 260 nm. (**C**) Comparison of isowighteone yield in the culture medium of hairy root cultures elicited for different time points. Yields are expressed in mg/L. Each bar represents the average of three biological replicates. Error bar represents standard deviation. Statistical analysis was performed using one-way ANOVA with Tukey’s multiple comparisons test. The lower-case letters above the column represent significant (between different letters) or non-significant (between the same letter) statistical differences. (Significance level between a and b, *p* < 0.05).

**Table 2:** Yield of isowighteone in the hairy root culture of pigeon pea (line G4). Twelve-day-old cultures were co-treated with methyl jasmonate, methyl-β-cyclodextrin, hydrogen peroxide, and magnesium chloride.

Source	Yield of Isowighteone (μg/g DW)	
	Control	Elicitor Treatment
Hairy root tissue	29.08 ± 3.7	346.35 ± 63.65
Culture medium	bDL ^a^	7712.27 ± 441.23
Total yield of isowighteone	29.08 ± 3.7	8058.618 ± 445.78

^a^ Below the detection limit.

**Figure 6:**
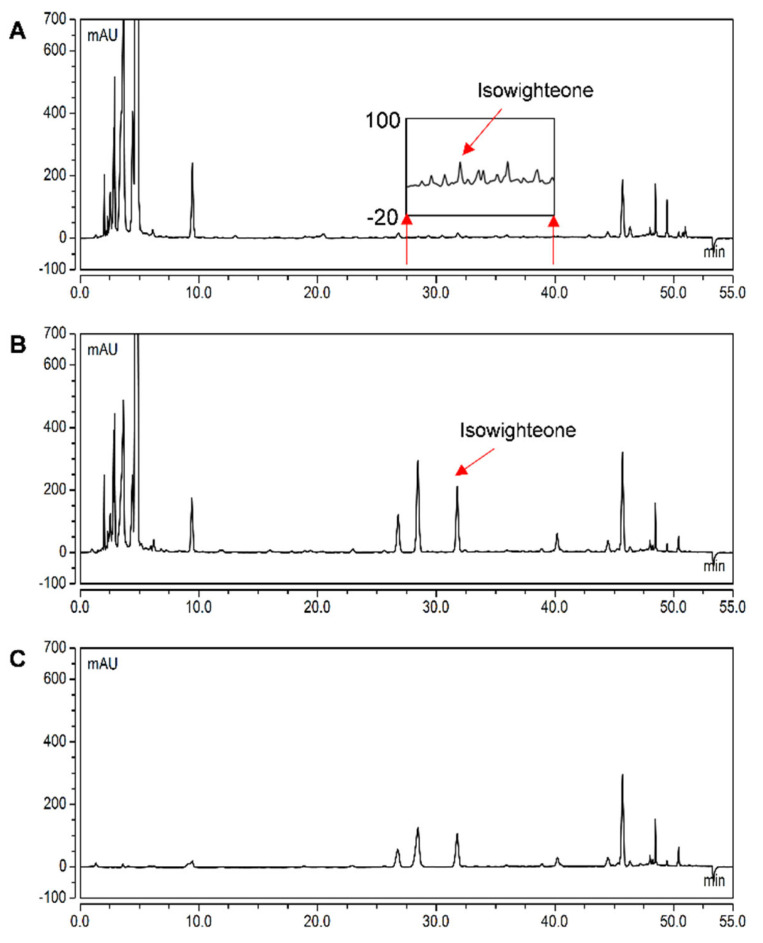
Comparison of isowighteone yield in hairy root tissues of 12-day-old pigeon pea hairy roots. HPLC chromatogram of (**A**) non-elicited hairy root tissue extracted using 70% ethanol; (**B**) 144 h elicited hairy roots extracted using 70% ethanol; and (**C**) 144 h elicited hairy roots extracted using ethyl acetate. All chromatograms were monitored at 260 nm.

The compound isowighteone was misidentified as cajaninstilbene acid due to an error in previous literature. Thus, the literature review related to cajaninstilbene acid has been removed or replaced with literature related to isowighteone. With this correction, the order of some references has been adjusted accordingly.

We also would like to change the first paragraph of Section 1. Introduction from:

Pigeon pea (*Cajanus cajan* (L.) Millsp.) is an important perennial legume crop with high medicinal and nutritional values. Extracts from pigeon pea leaves are rich in different flavonoids and stilbenoids which exhibit multiple therapeutic effects on inflammation, diabetes, dysentery, hepatitis, diarrhea, measles, and various other illnesses [1–3]. Among plant phenolics, the prenylated stilbenoid derivative cajaninstilbene acid (CSA) is one of the major bioactive metabolites in pigeon pea [4]. Specifically, CSA exhibits a neuroprotective function in vivo by activating the AMPK/Nrf2 pathway to reduce mitochondrial dysfunction [5]. CSA has also shown antibacterial activity against gram-positive bacteria including vancomycin-resistant *Enterococcus*, *Staphylococcus aureus*, and *Bacillus subtilis*, and antibiofilm activity against gram-negative bacteria including *Pseudomonas* aeruginosa [6–8]. In addition, CSA has demonstrated anti-inflammatory activity both in vivo and in vitro by inhibiting NF-κB and MAPK pathways [9]. Furthermore, studies suggest that CSA shows therapeutic potential in the treatment of both osteoporosis and the early stages of Alzheimer’s disease, with it also demonstrating antidepressant-like effects in mice models [10–12] and higher antioxidant activity than the stilbenoid resveratrol in cell-free systems [13]. The diverse biological properties of CSA support its potential application as a nutraceutical for human health benefits.

to:

Pigeon pea (*Cajanus cajan* (L.) Millsp.) is an important perennial legume crop with high medicinal and nutritional values. Extracts from pigeon pea leaves are rich in different flavonoids and stilbenoids which exhibit multiple therapeutic effects on inflammation, diabetes, dysentery, hepatitis, diarrhea, measles, and various other illnesses [1–3]. Several flavonoids have been reported from extracts of pigeon pea including apigenin, luteolin, isorhamnetin, vitexin, isovitexin, orientin, pinostrobin, isowighteone, and quercetin [4–6]. Among these flavonoids, isowighteone (3’-isoprenyl genistein) has been only reported from seedlings of pigeon pea treated with fungus and silver nitrate solution previously [5,6]. Isowighteone has shown antibacterial activity against gram-positive bacteria including *Listeria monocytogenes*, methicillin-resistant *Staphylococcus aureus*, methicillin-sensitive *Staphylococcus aureus*, and gram-negative bacteria including *Escherichia coli* [7,8]. Additionally, isowighteone has shown higher cytotoxicity against human colon carcinomas, potential pro-apoptotic property, and anti-inflammatory activities [9,10].

Moreover, the name of the compound cajaninstilbene acid in the whole text has been replaced with isowighteone. Correction has also been made to the Supplementary Materials.

The authors apologize for any inconvenience caused and state that the scientific conclusions are unaffected. This correction was approved by the Academic Editor. The original publication has also been updated.
