# Supplementary material for: Production and Secretion of Isowighteone in Hairy Root Cultures of Pigeon Pea (Cajanus cajan) Co-Treated with Multiple Elicitors
Source: Plants (Basel). 2022 Mar 21;11(6):834. doi: 10.3390/plants11060834 (PMC8951554; doi:10.3390/plants11060834)
Supplement: Supplementary file 1 [file plants-11-00834-s001.zip › plants-1618384-supplementary.pdf]

Supplementary Information

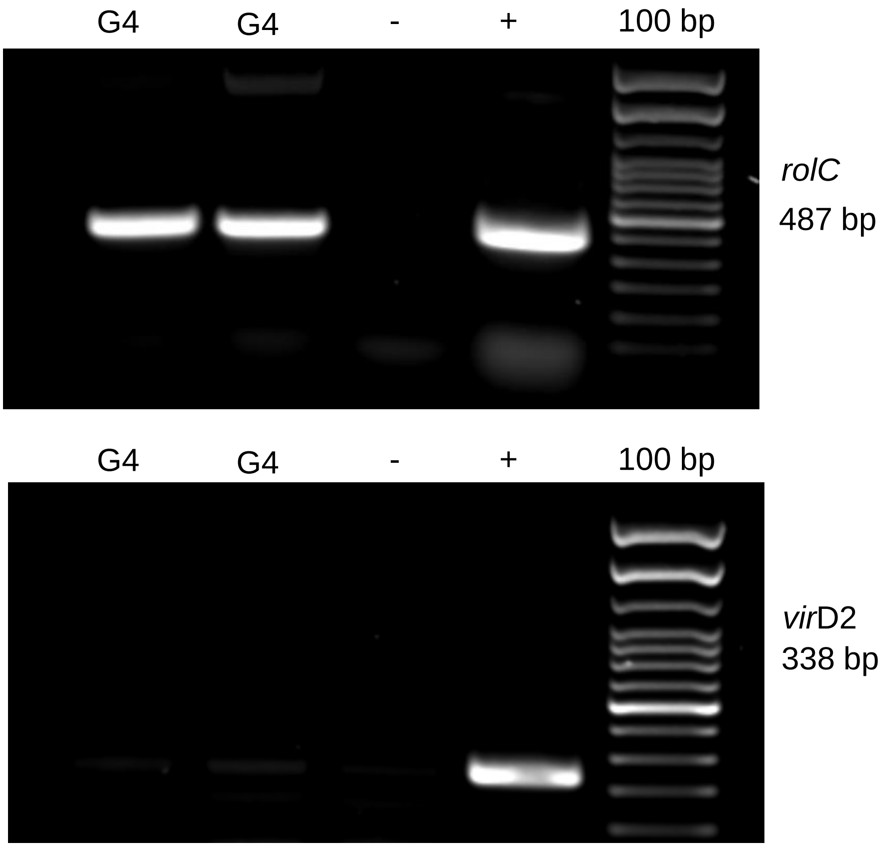

Figure S1. Molecular analysis by PCR of hairy roots of pigeon pea (line G4). Primers targeting the *aux1*, *rolC* and *virD2* genes were used.Plasmid pRiK599 was used as positive control and ddH_2O was used as negative control.

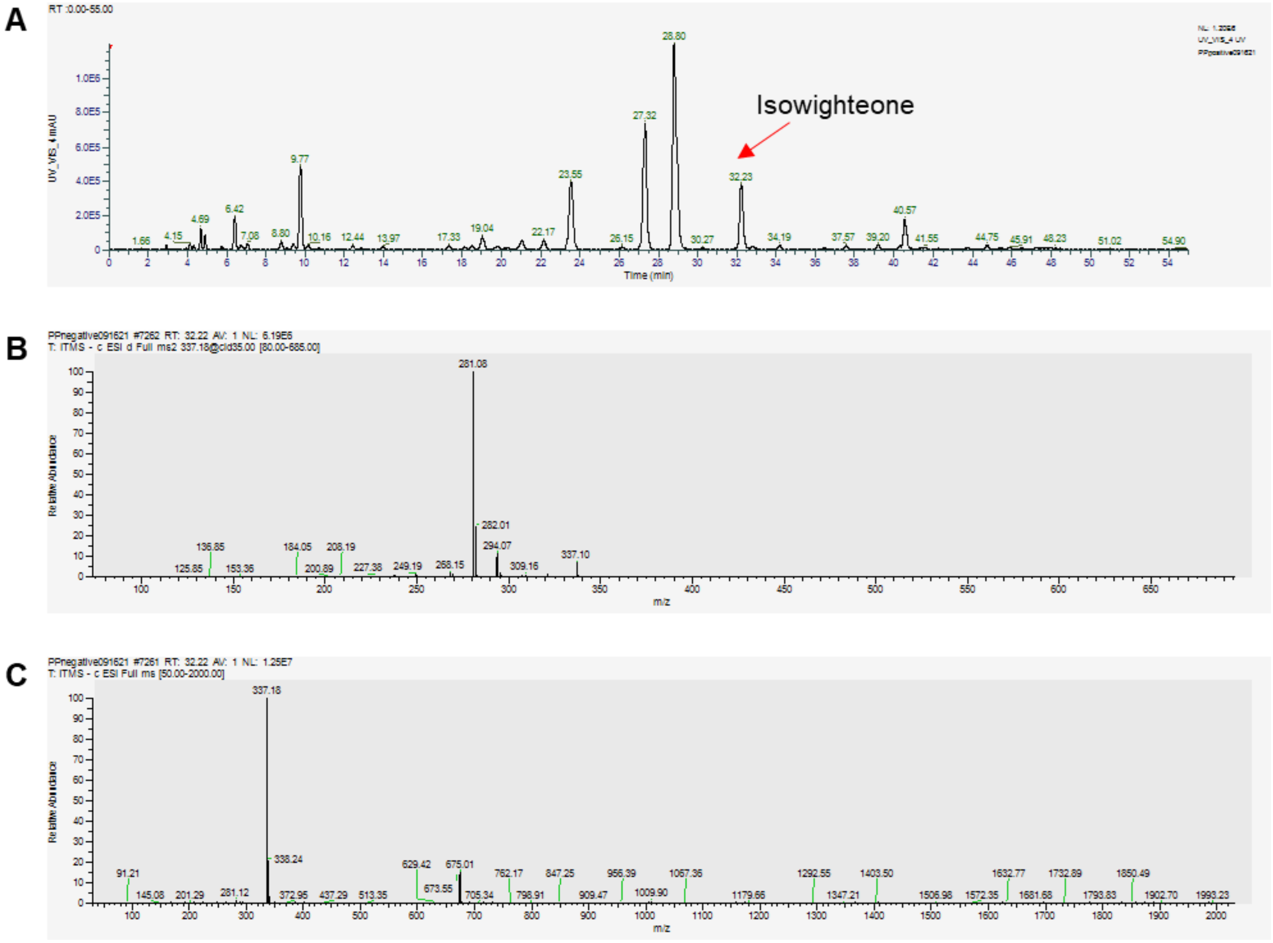

Figure S2. LC-MS analysis of Isowighteone under negative mode. (A) HPLC chromatogram of ethyl acetate extract of pigeon pea; (B) MS ion chromatogram; (C) MS^2 ion chromatogram
